# Kidney Beam-A Cost-Effective Digital Intervention to Improve Mental Health

**DOI:** 10.1016/j.ekir.2024.08.030

**Published:** 2024-09-02

**Authors:** Sharlene A. Greenwood, Juliet Briggs, Christy Walklin, Emmanuel Mangahis, Hannah M.L. Young, Ellen M. Castle, Roseanne E. Billany, Elham Asgari, Sunil Bhandari, Nicolette Bishop, Kate Bramham, James O. Burton, Jackie Campbell, Joseph Chilcot, Nicola Cooper, Vashist Deelchand, Matthew P.M. Graham-Brown, Lynda Haggis, Alexander Hamilton, Mark Jesky, Philip A. Kalra, Pelagia Koufaki, Kieran McCafferty, Andrew C. Nixon, Helen Noble, Zoe L. Saynor, Maarten W. Taal, James Tollitt, David C. Wheeler, Thomas J. Wilkinson, Hannah Worboys, Jamie Macdonald

**Affiliations:** 1 Renal Therapies Department, King’s College Hospital, London, UK; 2 Centre for Nephrology, Urology and Transplantation, Faculty of Life Sciences, King’s College London, London, UK; 3 Leicester Diabetes Centre, University Hospitals of Leicester NHS Trust, Leicester, UK; 4 Diabetes Research Centre, University of Leicester, Leicester, UK; 5 School of Physiotherapy, Department of Health Sciences, Brunel University, London, UK; 6 Department of Cardiovascular Sciences, University of Leicester, Leicester, UK; 7 Department of Nephrology, Guys and St Thomas’s Hospital, London, UK; 8 Department of Nephrology, Hull University Teaching Hospitals NHS Trust, Hull, UK; 9 School of Sport, Exercise and Health Sciences, Loughborough University, Loughborough, UK; 10 Faculty of Health, Education and Society, University of Northampton, Northampton, UK; 11 Department of Psychology, Psychology and Neuroscience, King’s College London, London, UK; 12 Department of Population Health Science, University of Leicester, Leicester, UK; 13 Department of Nephrology Royal Free Hospital, London, UK; 14 Dept of Nephrology, Royal Devon and Exeter NHS Foundation Trust, Exeter, UK; 15 Department of Nephrology, Nottingham NHS Trust, Nottingham, UK; 16 Department of Nephrology Royal Hospital, Northern Care Alliance NHS Foundation Trust, Salford, UK; 17 Dietetics, Nutrition and Biological Sciences, Physiotherapy, Podiatry and Radiography Division, Queen Margaret University, Edinburgh, UK; 18 Department of Nephrology, Barts Health NHS Trust, London, UK; 19 Department of Renal Medicine, Lancashire Teaching Hospitals NHS Foundation Trust, Preston, UK; 20 Division of Cardiovascular Sciences, The University of Manchester, Manchester, UK; 21 School of Nursing and Midwifery, Queen’s University Belfast, Belfast, UK; 22 School of Health Sciences, University of Southampton, Southampton, UK; 23 Centre for Kidney Research and Innovation, School of Medicine, University of Nottingham, Nottingham, UK; 24 Department of Renal Medicine, University College London, London, UK; 25 National Institute of Health Research Leicester Biomedical Research Centre, Leicester, UK; 26 Institute for Applied Human Physiology, Bangor University, Bangor, UK

**Keywords:** chronic kidney disease, cost-effectiveness, digital health intervention, physical activity, quality of life

## Abstract

**Introduction:**

There is inequity in the provision of physical rehabilitation services for people living with chronic kidney disease (CKD). The Kidney BEAM trial evaluated the clinical value and cost effectiveness of a physical activity digital health intervention (DHI) in CKD.

**Methods:**

In a single-blind, 11 center, randomized controlled trial, 340 adult participants with CKD were randomly assigned to either the Kidney BEAM physical activity DHI or a waitlist control. This study assessed the difference in the Kidney Disease Quality of Life Short Form 1.3 Mental Component Summary (KDQoL-SF1.3 MCS) between intervention and control groups at 6-months, and cost-effectiveness of the intervention.

**Results:**

At 6-months, there was a significant difference in mean adjusted change in KDQoL MCS score between Kidney BEAM and waitlist control (intention-to-treat adjusted mean: 5.9 [95% confidence interval, CI: 4.4–7.5] arbitrary units [AU], *P* < 0.0001), and a 93% and 98% chance of the intervention being cost-effective at a willingness-to-pay threshold of £20,000 and £30,000 per quality-adjusted life year gained.

**Conclusion:**

The Kidney BEAM physical activity DHI is a clinically valuable and cost-effective means to improve mental health-related quality of life (HRQoL) in people with CKD (trial registration no. NCT04872933).

## Graphical abstract

**Figure:**
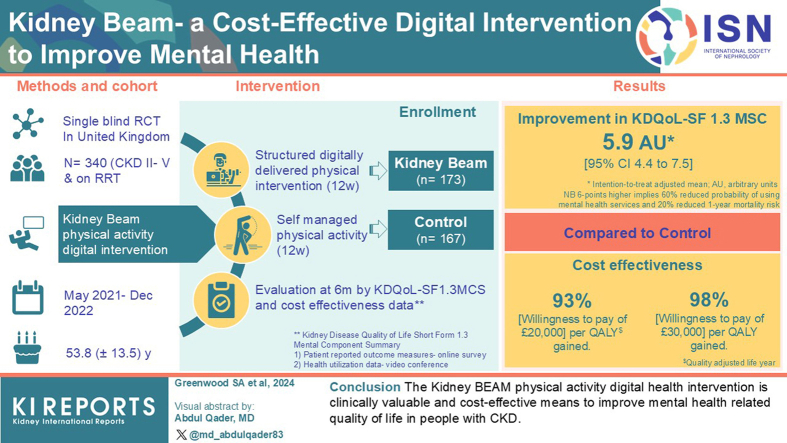

CKD affects more than 10% of the adult population worldwide, amounting to >800 million individuals, and is predicted to be the fifth highest cause of years of life lost worldwide by 2040.^1^ Physical inactivity is the fourth leading risk factor for global mortality, is a major risk factor for multimorbidity in people with chronic disease and has been associated with poor mental HRQoL.^2^^,^^3^ Consequently, interventions to enhance physical activity, mental health and HRQoL are of global interest and have been the focus of disease-specific guidelines, including those for people living with CKD.^4^, ^5^, ^6^

Although there may be benefits to in-person kidney rehabilitation,^7^ this has not been provided routinely in the UK,^8^ and policy-related barriers restrict access to exercise provision globally, leading to health inequality.^9^ One of the barriers to implementation has been a dearth of cost-effectiveness data to support the adoption of kidney-specific physical rehabilitation programs into already financially stretched health care systems.^10^ Even where there has been evidence published, such as the results from a UK study that reported the cost-effectiveness of intra-dialytic cycling programs,^11^ further complexities around availability of exercise personnel, equipment and unit-level support have resulted in little meaningful adoption to date.^10^ In addition, physical activity and exercise training trials in this patient population often neglect to report on whether there are sustained benefits from structured physical activity interventions, questioning the longer-term benefit and cost efficiency of these interventions when considering commissioning. We have anticipated these requirements by providing the 6-month patient outcome and health care utilization analyses reported here within.

The importance of DHIs has been highlighted in the World Health Organization global strategy on digital health 2020 to 2025.^12^ Furthermore, the utilization of DHIs can activate patients to engage in online lifestyle interventions and education, which can promote self-management and improve health outcomes for those with chronic disease.^13^

The 12-week Kidney BEAM physical activity DHI demonstrated clinically meaningful and statistically significant improvements in mental HRQoL, physical function, and patient activation (the ability to self-manage health behaviors) for people living with CKD,^14^ strongly supporting the efficacy of physical activity DHIs in the short-term. However, the Transtheoretical Model suggests that maintenance of a behavior can only be assumed if sustained for at least 6-months.^15^ Therefore, we hypothesized that 6-months of a physical activity DHI would reveal clinically meaningful improvements in mental HRQoL and be a cost-effective solution to deliver physical activity interventions for people living with CKD. The trial was codesigned with people with lived experience and targeted mental HRQoL because this was the most important outcome to the patients who we consulted with. Quality of life and life participation have been highlighted by the SONG initiative as being important to people living with CKD across the disease trajectory.^16^

## Methods

### Study Design

The 6-month Kidney BEAM Trial was a multicenter, randomized, single-blind, controlled waitlist trial to assess the clinical value and cost-effectiveness of a physical activity DHI on HRQoL in people with CKD that was conducted at eleven centers in the UK. The trial design, protocol, and baseline characteristics of the participants have been published previously,^17^^,^^18^ as have the 12-week results of the Kidney Beam Trial.^14^ The protocol was approved by the UK Bromley Research Ethics Committee at King’s College Hospital National Health Service (NHS) Trust, London, UK. The trial was designed and overseen by a trial steering committee and a data monitoring committee.

### Participants

Adults with established CKD, including those who were predialysis (CKD stages 2–4) and those on kidney replacement therapy (dialysis and kidney transplantation), were eligible for a DHI if they had access to a digital device and wi-fi connectivity. Recruitment occurred at kidney centers across England, UK, intentionally chosen to represent the geographical diversity of the UK CKD population. Potential participants underwent screening, and their clinical records were reviewed to confirm eligibility. Trained research staff approached suitable adults face-to-face during clinic visits or through telephone. Exclusions included self-reported participation in a recent exercise program or use of a physical activity DHI within the last 3-months, persistent uncontrolled hypertension, unstable angina, and conditions preventing engagement in a physical activity intervention, such as peripheral vascular or musculoskeletal diseases. Decisions to exclude participants based on the severity of peripheral vascular or musculoskeletal disease were adjudicated by the study team to prevent risk to the patient rather than an exclusion based on chart diagnosis alone. Informed written consent was obtained from all participants, and a detailed list of inclusion and exclusion criteria can be found in the methods paper.^17^

### Randomization and Masking

Participants were randomly assigned in a 1:1 ratio to the Kidney BEAM intervention group or the waitlist control group. Randomization was performed with the use of a web-based system, in randomly permuted blocks of 6. Randomization and treatment allocation were performed by an independent member of the research team and the allocation list was stored in a password-protected database. Given the nature of the intervention, it was not possible to blind the health care professionals providing the program or the participants. Outcome assessors were, however, blinded to treatment allocation. The statistical analysis plan and the health economic analysis plan^17^ were developed *a priori* by an independent statistician and health economist and were approved by the trial steering committee. Data entry and quality assurance were undertaken by data entry clerks unaware of treatment allocation. Data cleaning and analysis of outcome data were conducted by the independent statistician and health economist unaware of treatment allocation.

### Outcomes

The primary objective for this 6-month trial was to evaluate the change in the Kidney Disease Quality of Life Short Form 1.3 MCS between baseline and 24 weeks and to assess cost effectiveness. The MCS is composed of all scales of the SF-36 but is more heavily weighted to the vitality (energy/fatigue), social functioning, role emotional and mental health subscales of the KDQoL questionnaire. Secondary objectives included evaluating changes in the KDQoL-SF1.3 Physical Component Score at 24 weeks (which is more heavily weighted to the physical functioning, role-physical, bodily pain, general health subscales), other KDQoL subscales, the European Quality of Life 5-dimension, 5-level questionnaire (converted to the European Quality of Life 5-dimension, 3-level to allow comparison with UK normative data) and health care utilization data. All outcome measures were chosen as valid and reliable tools to measure the primary and secondary outcomes in this patient population.^19^ All patient-reported outcome measures were completed via an online survey. Health utilization data was also obtained via video conference with participants. Safety outcomes were based on adverse-event reporting. An independent data monitoring committee had oversight of trial safety.

### Health Care Utilization

Data on associated hospital costs, primary care consultations, and social care usage were collected via patient interview for the pretrial and within trial period. Prescribed medication costs were collected from hospital records. Intervention costs assume a cost of £15/participant/yr and consisted of physiotherapy time, physiotherapy assistant time, and running costs for the Kidney BEAM platform. One experienced physiotherapy assistant at whole time (1.0 whole time equivalent), and 1 senior, experienced physiotherapist at 10% of their whole time (0.1 whole time equivalent) per 340 participants were costed in at current NHS staff salary rates.^20^ This intervention cost reflects a proposed population-based contract assuming a 10% sign-up rate to the intervention across the CKD population of England. Resources were valued using national tariffs.^21^^,^^22^ All costs were expressed in 2021/2022 UK pounds (£) and inflated to this base year where appropriate using the UK Consumer Price Health Index.^20^

### Intervention

The 12-week structured physical activity intervention has been described in detail elsewhere.^14^^,^^19^ In brief, the 6-month Kidney BEAM intervention (https://beamfeelgood.com/home), which included a rolling 12-week structured digitally delivered physical activity intervention, was delivered by specialist kidney physiotherapists through “live” sessions, which were delivered in real-time via the digital platform, and a prerecorded on-demand kidney rehabilitation program, followed by 12 weeks of self-managed physical activity accessed through the Kidney BEAM platform. The structured 12-week sessions comprised a 10-minute warm-up and cool-down involving general upper and lower limb mobility and stretching. The core session included 20 to 30 minutes of moderate-intensity aerobic and resistance exercises, delivered both in a standing and seated position. In addition, participants received 15 minutes of disease-specific education on topics related to managing kidney health, such as managing a kidney diet and understanding diabetes, weekly. A physiotherapy assistant, trained in motivational interviewing, provided ongoing general encouragement through weekly telephone or email communication. Participants could review their progress through their personalized dashboard on the platform. After completing the 12-week program and assessing outcomes, participants in the intervention group were advised by the physiotherapy assistant to maintain self-management of their physical activity behavior with ongoing access to the Kidney BEAM platform. Participants who were allocated to the waitlist control group did not participate in a 12-week structured exercise program and were only sign-posted to Kidney BEAM after the 12-week assessment.

### Statistical Analysis

The trial was designed to detect a clinically meaningful 3 AU difference in HRQoL Kidney Disease Quality of Life Short Form 1.3 MCS score between groups at 12 weeks and 6-months. An estimated sample size of 106 participants in each group (*N* = 212) based on an MCS with a mean of 45 AU, (SD: 10 AU) and correlation between repeated measures of 0.7, would allow a clinically meaningful difference of 3 AU to be detected at 80% power and 5% alpha. Specifically, a 3-point difference in MCS is associated with an odds ratio of 1.13 for being unable to work or an odds ratio of 1.16 for 1-year job loss. The probability of using mental health services is increased by approximately 30% (odds ratio = 1.31), and there is a 30% increased risk of depression (odds ratio = 1.34). It is also associated with a 10% higher 1-year mortality risk (odds ratio–1.10). A total of 340 patients were included to allow for a 30% drop-out and to ensure power for secondary outcomes.^23^ The baseline characteristics were described using summary statistics.^17^ Primary and secondary outcomes at 6-months were analyzed with an analysis of covariance model, with baseline data and age as covariates. Independence of covariates and approximated normality of residuals were confirmed for all analyses. All analyses were performed in the intention-to-treat population using a last observation carried forward approach to missing data because this gives the most conservative result. The results from the last observation carried forward analysis for the primary outcome were compared to those from a multiple imputation sensitivity analysis using pooled results from 5 linear regression imputations. Per protocol analyses in which only cases with observations at both baseline and week 24 were included, were also completed to assess efficacy under ideal conditions. Two-sided *P* values of less than 0.05 were considered to indicate statistical significance. Analyses were performed with SPSS (version 28, IBM, NY).

The reporting of the Health Economic Analysis adheres to the CHEERS 2022 Checklist.^24^ The within-trial economic analyses were performed using individual patient level data collected from the trial. The base case analysis included all participants completing the 12 week and 6-month follow-up with missing resource use items imputed using a last value carried forward approach. Area under the curve methods were used to calculate the quality-adjusted life years (QALYs) accrued by each person during the intervention period based on the European Quality of Life 5-dimension, 5-level cost utility data collected at baseline and at 3 and 6-months. The trial was conducted in the UK, which has an NHS providing publicly funded health care, primarily free of charge at the point of use. The primary economic analysis was from the NHS and personal social services perspective. The primary economic analysis compared the costs and consequences of each arm over the 6-months following randomization. For the analysis, we adopted a bivariate model for estimating incremental costs and effects in WinBUGS using Markov Chain Monte Carlo simulation methods^19^ with costs and 1-QALYs expressed as Gamma distributions. Bayesian methods require the specification of prior distributions for parameters of the distributions. Here, we used prior distributions intended to be noninformative, because we wanted the resulting inferences to only depend on the data. For the base-case analysis, the bivariate model incorporated adjustment for baseline costs (12 weeks prior to intervention) and European Quality of Life 5-dimension to allow for imbalance between the groups using the methods proposed by Nixon and Thompson 2005.^25^ Posterior distributions of the parameters of interest for the inferences about cost-effectiveness were derived from 20,000 iterations of the Markov chain, after an initial 20,000 iterations were discarded to ensure convergence. Results were expressed in terms of cost per QALY gained (i.e., the incremental cost-effectiveness ratio), which was estimated for the Kidney BEAM group compared with the waitlist control group.

### Inclusion and Ethics

The trial was designed and overseen by a trial steering committee and a data monitoring committee. The protocol and related documents were approved by Bromley NHS Research Ethics Committee (REC) (21/LO/0243) and the Health Research Authority and was prospectively registered (NCT04872933) on May 5, 2021. All methods were carried out in accordance with relevant guidelines and regulations. Informed consent was obtained from all subjects and/or their legal guardian(s).

## Results

### Participants

**Figure 1:**
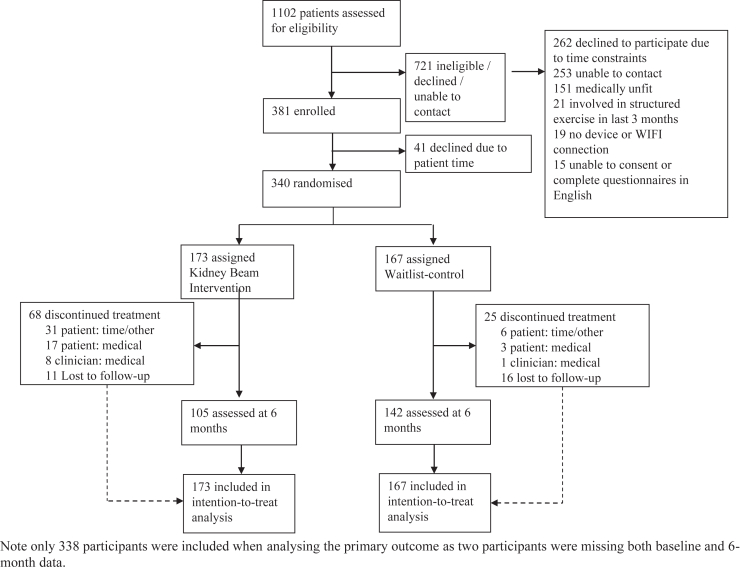
Flowchart of participants through the trial.

From May 6, 2021, to October 30, 2022, 1102 people were assessed for eligibility (Figure 1). After excluding 721 people (65%), 381 (35%) participants were consented and a total of 340 participants (31%) from 11 centers attended a baseline visit. The 2 main reasons for not engaging with the trial were time constraints associated with the research trial and potential participants that passed screening but were not able to be contacted to consent and participate in the trial. One-hundred seventy-three people (51%) were randomly assigned to the Kidney BEAM intervention group, and 167 (49%) were assigned to the waitlist control group. Of these, 247 (73%) participants completed the 6-month trial: 105 in the intervention group (61% of those randomized) and 142 in the waitlist control group (85% of those randomized). All 340 participants were included in the intention-to-treat analysis. Overall, the 2 groups were generally well-balanced with respect to baseline characteristics (Table 1), albeit the mean European Quality of Life 5-dimension-3 level utility scores were lower in the intervention group and there was more self-reported burden of kidney disease, pain, and sexual dysfunction in the intervention group (Table 2).

**Table 1:** Baseline demographic data

Variable	*n*	All	*n*	Kidney BEAM	*n*	Waitlist control
Age (yr) (SD)	340	53.8 (13.5)	173	53.9 (13.6)	167	53.8 (13.5)
Sex (*n*) (%)	340		173		167	
Male		185 (54)		96 (55)		89 (53)
Female		155 (46)		77 (45)		78 (47)
Ethnicity (*n*) (%)	339		173		166	
Black		39 (11.5)		20 (11.6)		19 (11.4)
White		254 (74.9)		127 (73.4)		127 (76.5)
Asian		39 (11.5)		22 (12.7)		17 (10.2)
Biracial		7 (2.1)		4 (2.3)		3 (2.1)
Body mass index (kg/m^2^) (IQR)	327	28.4 (24.8–33.3)	165	27.9 (24.7–33.4)	162	28.8 (24.9–33.0)
Smoking (*n*) (%)	339		172		167	
Current		16 (4.7)		5 (2.9)		11 (6.6)
Former		130 (38.3)		77 (44.8)		53 (31.7)
Never		193 (56.9)		90 (52.3)		103 (61.7)
Alcohol consumption (*n*) (%)	339		172		167	
More than recommended		26 (7.7)		14 (8.1)		12 (7.2)
Less than recommended		174 (51.3)		89 (51.7)		85 (50.9)
Nondrinker		139 (41.0)		69 (40.1)		70 (41.9)
Blood pressure (mm Hg) (SD)	307		154		153	
SBP		136.5 (18.4)		135.3 (19.3)		137.8 (17.5)
DBP		79.7 (10.7)		78.6 (11.1)		80.7 (10.2)
Resting heart rate (bpm) (SD)	207	77.6 (14.7)	103	77.8 (14.6)	104	77.3 (14.8)
Medical History (n) (%)	340		173		167	
CVA		8 (2.4)		4 (2.4)		4 (2.4)
MI		8 (2.4)		3 (1.7)		5 (3)
Diabetes		76 (22.4)		37 (21.4)		39 (23.4)
Hypertension		235 (69.1)		115 (68.9)		120 (69.4)
Cause of kidney disease (*n*) (%)	340		173		167	
Diabetic nephropathy		31 (9.1)		13 (7.5)		18 (10.8)
Hypertension		38 (11.2)		21 (12.1)		17 (10.2)
Nephrosclerosis		1 (0.3)		1 (0.6)		0 (0)
IgA nephropathy		39 (11.5)		18 (10.4)		21 (12.6)
Tubulointerstitial nephritis		5 (1.5)		2 (1.2)		3 (1.8)
PKD		60 (17.6)		31 (17.9)		29 (17.4)
Obstructive nephropathy		7 (2.1)		2 (1.2)		5 (3)
Medullary sponge kidney disease		0 (0)		0 (0)		0 (0)
Membranous nephropathy		5 (1.5)		5 (2.9)		0 (0)
Lupus nephritis		5 (1.5)		4 (2.3)		1 (0.6)
Unknown		65 (19.1)		33 (19.1)		32 (19.2)
Other		84 (24.7)		43 (24.9)		41 (24.6)
CKD stage (%)	339		172		167	
Stage 2		55 (16.2)		27 (15.7)		28 (16.8)
Stage 3A		62 (18.3)		29 (16.9)		33 (19.8)
Stage 3B		76 (22.4)		45 (26.2)		31 (18.6)
Stage 4		67 (19.8)		34 (19.8)		33 (19.8)
Stage 5		79 (23.3)		37 (21.5)		42 (25.1)
Treatment modality (*n*) (%)	340		173		167	
Non-dialysis dependent kidney disease		160 (47)		75 (43)		85 (51)
Kidney transplant recipient		118 (35)		65 (38)		53 (32)
Dialysis therapy		62 (18)		33 (19)		29 (17)
HbA1c (mmol/mol)	124	39 (35–48)	64	39 (34–50)	60	39 (36–47)
Creatinine (μmol/l)	332	159 (106–293)	170	159 (109–279)	162	161 (106–330)
CRP (mg/l)	169	4 (2–9)	92	3.9 (2–10)	77	4 (2–9)

CKD, chronic kidney disease; CRP, C-reactive protein; CVA, cerebrovascular accident; DBP, diastolic blood pressure; HbA1C, glycated hemoglobin; IQR, inter-quartile range; Kidney BEAM, Kidney BEAM intervention group (physical activity training and education plus usual care); MI, myocardial infarction; *n*, total number of available data; PKD, polycystic kidney disease; SBP, systolic blood pressure; waitlist control, waitlist control group.

Data are mean (SD), median (IQR), or number (%), as appropriate.

**Table 2:** Response of primary and secondary outcome measures to the Kidney BEAM intervention (intention to treat analysis)

Outcome measure	*n*	Baseline mean (SD)	6 mo mean (SD)	Mean difference in change between groups (Kidney BEAM - waitlist control) mean (95% CI)	*P* value	Observed power
Primary outcome						
KDQoL MCS (AU)						
Kidney BEAM	171	44.6 (10.8)	48.7 (10.5)	5.9 (4.4–7.5)	<.0001	1.00
Waitlist control	167	48.1 (10.5)	43.5 (10.3)			
Secondary outcomes						
KDQOL PCS (AU)						
Kidney BEAM	171	40.0 (11.7)	42.9 (11.02)	1.5 (−0.03 to 2.9)	0.055	0.48
Waitlist control	167	41.3 (11.2)	42.5 (11.3)			
Symptom problem list						
Kidney BEAM	140	76.6 (18.2)	77.8 (17.9)	0.6 (−2.2 to 3.3)	0.67	0.07
Waitlist control	143	79.9 (16.8)	79.7 (18.7)			
Effects of Kidney Disease						
Kidney BEAM	166	69.1 (26.5)	72.3 (26.1)	1.0 (−2.8 to 4.9)	0.59	0.08
Waitlist control	161	75.6 (23.6)	76.3 (26.2)			
Burden of kidney disease						
Kidney BEAM	172	55.1 (31.2)	61.7 (30.7)	5.3 (2.0–8.6)	0.0017	0.88
Waitlist control	167	64.9 (30.5)	64.7 (29.9)			
Work status						
Kidney BEAM	84	61.8 (40.6)	61.2 (38.1)	−5.2 (−12.3 to 2.0)	0.15	0.29
Waitlist control	120	61.7 (41.4)	65.8 (37.8)			
Cognitive function						
Kidney BEAM	172	74.7 (19.3)	78.5 (17.9)	2.3 (−0.3 to 4.9)	0.082	0.41
Waitlist control	167	78.7 (19.5)	78.5 (17.9)			
Quality of social interaction						
Kidney BEAM	172	72.0 (18.9)	76.9 (17.7)	7.1 (4.1–10.0)	<.0001	1.00
Waitlist control	167	73.6 (18.2)	70.8 (18.7)			
Sexual function						
Kidney BEAM	102	42.3 (41.6)	41.5 (41.1)	−3.4 (−11.7 to 5.0)	0.427	0.124
Waitlist control	102	48.5 (41.7)	49.1 (43.4)			
Sleep						
Kidney BEAM	171	55.6 (19.5)	60.6 (18.7)	6.5 (3.5–9.5)	<.0001	0.99
Waitlist control	166	57.7 (20.3)	55.7 (21.0)			
Social support						
Kidney BEAM	158	72.7 (27.6)	77.0 (25.7)	4.0 (−1.0 to 9.0)	0.117	0.35
Waitlist control	150	75.7 (28.3)	74.7 (28.7)			
Dialysis staff encouragement						
Kidney BEAM	77	78.7 (24.3)	75.8 (26.3)	−6.1 (−12.2 to −0.03)	0.049	0.51
Waitlist control	68	77.2 (27.4)	80.7 (27.3)			
Overall health						
Kidney BEAM	85	60.1 (19.9)	62.6 (18.0)	−1.3 (−5.5 to 2.9)	0.55	0.09
Waitlist control	118	58.1 (18.1)	62.5 (20.1)			
Patient satisfaction						
Kidney BEAM	93	73.5 (22.8)	75.6 (21.2)	1.8 (−2.6 to 6.3)	0.417	0.128
Waitlist control	87	73.7 (24.3)	74.1 (22.4))			
Physical functioning						
Kidney BEAM	171	60.9 (30.1)	68.0 (28.2)	6.29 (2.9–9.7)	0.0003	0.95
Waitlist control	167	64.2 (30.7)	64.3 (30.5)			
Role physical						
Kidney BEAM	171	48.1 (41.8)	62.9 (42.6)	9.1 (1.8–16.3)	0.014	0.69
Waitlist control	167	51.0 (43.4)	55.4 (44.3)			
Pain						
Kidney BEAM	172	61.1 (26.4)	66.7 (26.0)	8.0 (3.8–12.2)	0.0002	0.96
Waitlist control	167	67.8 (27.7)	63.6 (29.8)			
General health						
Kidney BEAM	171	40.3 (21.6)	45.1 (22.2)	4.3 (1.6–7.0)	0.0018	0.88
Waitlist control	167	42.7 (21.6)	42.7 (22.0)			
Emotional wellbeing						
Kidney BEAM	171	67.0 (20.5)	74.3 (20.2)	4.0 (−1.0 to 9.0)	<0.0001	0.35
Waitlist control	167	70.3 (18.7)	65.9 (19.6)			
Role emotional						
Kidney BEAM	171	60.5 (42.5)	72.1 (39.4)	10.7 (3.1–18.4)	0.0058	0.79
Waitlist control	166	63.2 (42.3)	55.9 (43.7)			
Social function						
Kidney BEAM	172	61.6 (27.6)	69.4 (27.9)	10.1 (6.3–13.8)	<0.0001	1.00
Waitlist control	167	64.3 (30.2)	61.3 (28.9)			
Energy/fatigue						
Kidney BEAM	171	42.6 (21.4)	53.1 (23.1)	15.5 (12.6–18.4)	<0.0001	1.00
Waitlist control	167	45.0 (23.3)	39.5 (22.6)			
EQ-5D-3L utility score						
Kidney BEAM	171	0.65 (0.25)	0.71 (0.25)	0.10 (0.07–0.13)	<0.0001	1.00
Waitlist control	167	0.73 (0.23)	0.68 (0.26)			

ANCOVA, analysis of covariance; AU, arbitrary units; CI, confidence interval; control, waitlist control group (usual care); EQ-5D-3L, EuroQol 5-dimension descriptive system; KDQOL, Kidney Disease Quality of Life Short Form (KDQOL-SF 1.3); Kidney BEAM, Kidney BEAM intervention group (physical activity training and education plus usual care); MCS, Mental Component Score; PCS, Physical Component Summary.

Data are mean (SD), median (interquartile range), or mean (95% confidence interval) ANCOVA adjusted scores.

### Participant Adherence

A median of 15 (interquartile range, IQR: 9–22) of the recommended 24 sessions of structured physical activity were completed by participants in the Kidney BEAM intervention group during the structured 12-week physical activity component, representing a median adherence rate of 63% (IQR: 38%–92%). Participants completed a median of 529 (IQR: 283–814) minutes of structured physical activity (video/session length × number of sessions), the equivalent of 44 min/wk. A median of 6 (IQR: 1–10) of the recommended 12 sessions of education were completed, representing a median adherence rate of 50% (IQR: 8%–83%). Sixty-five of 105 participants (62%) from the Kidney BEAM intervention group continued to use the Kidney BEAM platform to complete self-managed physical activity sessions after the 12-week assessment. Between 12 weeks and 6-months, participants in the Kidney BEAM group completed a median of 7 (IQR: 3–41) sessions of self-managed physical activity sessions on the platform and completed a median of 286 (IQR: 103–1792) minutes of self-managed physical activity through the platform. As per protocol, participants from the waitlist control group were informed at consent that they could access the Kidney BEAM platform following the 12-week assessment. This was not actively encouraged by the team and only 15 of 142 participants (11%) from the waitlist control group did choose to self-sign-up to the platform and complete self-managed physical activity sessions on the Kidney BEAM platform between 12 weeks and 6-months. Participants from the waitlist control group completed a median of 11 (IQR: 5–46) sessions of self-managed physical activity using the platform, and a median of 119 (IQR: 90.5–1822) minutes of self-managed physical activity using the platform.

### Primary Outcomes

Using the most conservative last observation carried forward approach, there was a clinically relevant and statistically significant improvement in the KDQoL SF 1.3 MCS score after 6- months in the Kidney BEAM group compared to the control group of 5.9 (95% CI: 4.4–7.5) AU (*P* < 0.0001) (Table 2). Sensitivity analysis confirmed this result, by using multiple imputation of the 6-month missing values, and 5 iterations of linear regression imputation, revealing a pooled mean difference of 5.8 (3.1–8.4) AU (*P* < 0.0001).

**Figure 2:**
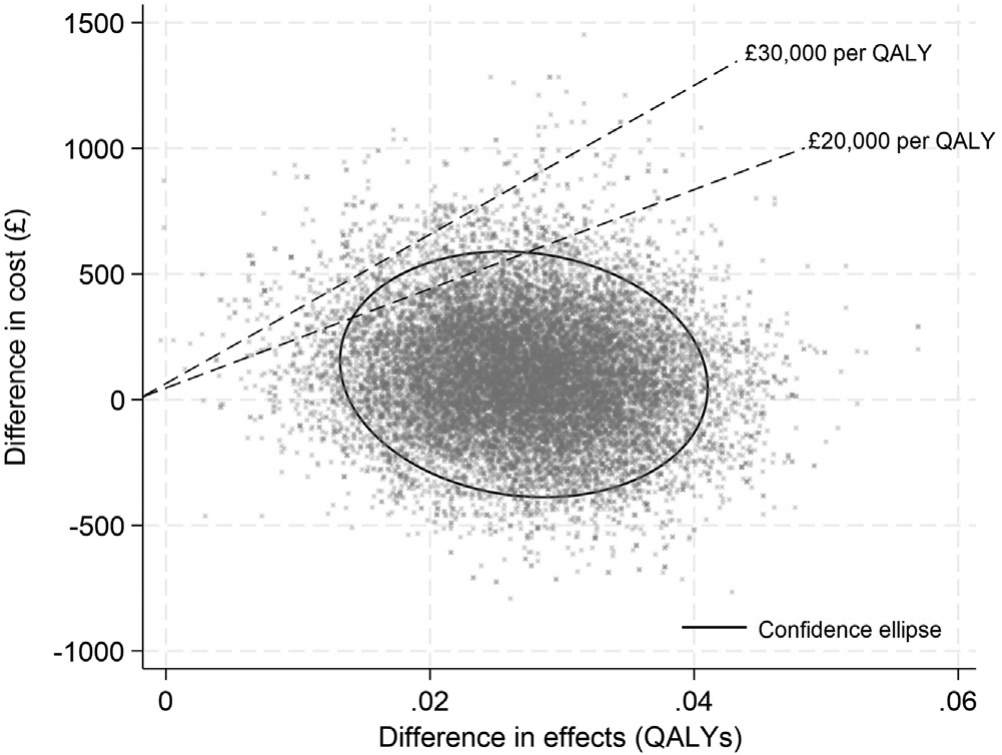
Cost-effectiveness plane with 95% confidence region. QALYs, quality-adjusted life years.

Regarding cost effectiveness, the adjusted intention-to-treat base case model, assuming a cost per participant of £15/yr, showed a mean cost saving of £93 (95% CI: −£360 to £613) per participant in health care utilization costs and a significant increment in QALYs of 0.027% (95% CI: 0.013%–0.040%) years per participant, resulting in a cost per QALY of £3446 for the Kidney BEAM intervention (Table 3 and Supplementary Table S1). This resulted in a 93% and 98% probability (indicated by the proportion of the ellipses below the willingness-to-pay threshold line, Figure 2) of the Kidney BEAM intervention being cost-effective, compared with waitlist control, at the willingness-to-pay thresholds of £20,000 and £30,000 per QALY gained, respectively (Figure 2 and Table 3). The adjusted complete-case model, assuming a cost per participant of £15/yr, showed a mean cost saving of £273.60 (95% CI: −£323 to £996.7) per participant in health care utilization costs and a significant increment in QALYs of 0.026 (95% CI: 0.009–0.043) years per participant, resulting in a cost per QALY of £10,523.08 for the Kidney BEAM intervention. This resulted in a 75% and 87% probability of the Kidney BEAM intervention being cost-effective, compared with waitlist control, at the willingness-to-pay thresholds of £20,000 and £30,000 per QALY gained (Figure 2). The significant increase in KDQoL MCS in the Kidney BEAM intervention group compared with waitlist control is associated with an incremental cost-effectiveness ratio of £14.44 per 1 unit change in KDQoL MCS (Supplementary Table S2). Exploratory analyses comparing the cost effectiveness of the Kidney BEAM DHI at varying costs per participant per year for the intervention (£30, £50, and £100) did not result in any change to the incremental cost-effectiveness ratio (Supplementary Table S3). Primary care, medication, hospital-associated, and total costs are presented by group at 12 weeks pretrial, and at 12 weeks and 6-months during the trial (Supplementary Tables S4 and S5).

**Table 3:** Base case model (assumes intervention £15/person/yr)

Variable	Base case model: LVCF for missing cost components adjusted for baseline costs and EQ-5D	Complete case analysis adjusted for baseline costs and EQ-5D
*n*: WL	132^a^	92^b^
*n*: KB	91^a^	66^b^
Mean difference in Cost	£93.03 (−£360.60 to £613.40)	£273.60 (−£323 to £996.7)
Mean difference in QALYs	0.027 (0.013–0.040)	0.026 (0.009–0.043)
Incremental cost-effectiveness ratio (ICER)	£3445.56	£10,523.08
Probability CE @ £20,000 per QALY gained	0.93	0.75
Probability CE @ £30,000 per QALY gained	0.98	0.87

Calculated at the average baseline value of cost (£1850) and EQ-5D score (0.70).

CE, cost-effectiveness; EQ-5D, EuroQol 5-dimension descriptive system; KB, Kidney BEAM; LVCF, last value carried forward; QALY, quality-adjusted life year; WL, waitlist.

a Excludes individuals with missing EQ-5D and cost baseline data (3 WL, 1 KB).

b Excludes individuals with missing EQ-5D and cost baseline data (1 WL, 1 KB).

### Secondary Outcomes

The change in the KDQoL MCS was primarily due to mean between-group improvements in the individual components of the KDQoL SF 1.3 questionnaire at the same time-point, including the social function, energy or fatigue, role emotional, and emotional wellbeing scales (Table 2).

Analysis of secondary outcomes revealed a significant improvement at 6-months in the European Quality of Life 5-dimension-3 level utility score of 0.10 (95% CI: 0.07–0.13) units (*P* < 0.0001) in favor of the Kidney BEAM group (Table 2). The mean between-group difference in the KDQoL Physical Component Score and the cognitive function subscale at 6- months were not significant (*P* = 0.055 and 0.082, respectively [Table 2]) but were significant on per protocol analysis (Supplementary Table S6). All other subscales revealed significant mean between-group differences at 6-months in favor of the intervention group (Table 2).

There were 9 unrelated serious adverse events recorded in a total of 9 of the 340 participants, with a similar incidence across both groups: 4 of the 9 (3%) in the Kidney BEAM group and 5 of the 9 (3%) in the control group across the 6-month trial period. There were no expected related or unrelated serious adverse events recorded in either group during the duration of the trial (Table 4).

**Table 4:** Number of patients with at least 1 serious adverse event by MedDRA system organ class during the Kidney BEAM Trial

Variable	All *n* (%)	Kidney BEAM *n* (%)	Waitlist control *n* (%)
Number of randomized patients who attended baseline visit	340	173	167
Number of patients with any event	9 (2)	4 (3)	5 (3)
Gastrointestinal disorders	2 (1)	1 (1)	1 (1)
Infections and infestations	4 (1)	2 (1)	2 (1)
Injury, poisoning, and procedural complications	2 (1)	1 (1)	1 (1)
Renal and urinary disorders	1 (0.3)	0 (0)	1 (1)

### Participant Dropouts and Missing Data

There was no obvious difference in participant characteristics between participants that completed the 6-month outcome assessment and participants that did not (Supplementary Table S7). Forty-seven of the 68 participants (77%) that did not complete the trial in the intervention group withdrew within the first week postbaseline assessment due to time constraints. As expected, the number of missing data points for the cost-effectiveness analyses increased as the trial progressed; however, at 6-months there were still 229 data points available for analysis (Kidney BEAM intervention group: *n* = 93; control group *n* = 136) (Supplementary Table S8).

## Discussion

The results from this 6-month trial demonstrate that the Kidney BEAM physical activity DHI resulted in a clinically meaningful, sustained improvement in mental HRQoL in people with CKD and was cost-effective. Our data will support commissioning of the Kidney BEAM innovation within the National Health System and inform commissioning of similar services in other health care systems.

Interventions that afford improvements in mental HRQoL are important for all people living with CKD, and may be particularly important for those people receiving dialysis therapy where lower levels of HRQoL have been associated with morbidity and mortality, and where every 1-point increase in MCS has been associated with a 2% reduction in the relative risk of death and a 1% reduction in the relative risk of hospitalization.^26^ Specifically, a 3-point difference in MCS is associated with an odds ratio of 1.13 for being unable to work or an odds ratio of 1.16 for 1-year job loss. The probability of using mental health services is increased by approximately 30% (odds ratio = 1.31), and there is a 30% increased risk of depression (odds ratio = 1.34). It is also associated with a 10% higher 1-year mortality risk (odds ratio = 1.10).^23^

The continued improvements in mental HRQoL determinants resulting from the 6-month Kidney BEAM intervention in the intention-to-treat analysis, were accompanied by an increase in physical HRQoL determinants that were not observed at the 12-week assessment point. Mean KDQoL Physical Component Score scores in the intervention group increased (*P* = 0.055 in intention-to-treat; *P* < 0.0001 in per protocol analysis) and were driven by improvements in the subscales of the KDQoL questionnaire that make up the composite score; including significant improvements in scores in the intention-to-treat population in role physical, physical functioning, pain, and general health. It is postulated that the perception of being able to complete, participate, and be confident in undertaking physical tasks may require an initial improved psychological perspective and the physiological gain in physical function associated with an initial supervised program, to achieve longer term gains in perception of physical well-being. A structured physical activity program as a “kick-start” precursor to physical HRQoL improvements, consolidated with a further 12 weeks of self-managed physical activity behavior appears to be essential to realize important physical HRQoL gains in a patient population where high levels of sedentary behavior are common and the role of exercise counselling to improve both mental and physical health outcomes is far from routine in kidney care management.^10^

This trial revealed that the Kidney BEAM 6-month physical activity DHI, specifically designed for people living with CKD, significantly improved mental HRQoL compared with waitlist control with a 93% and 98% chance of the Kidney BEAM intervention being cost-effective compared to waitlist control at a willingness-to-pay of £20,000 and £30,000 per QALY gained. Every increment in QALYs resulting from a 6-month program of Kidney BEAM is associated with an incremental cost-effectiveness ratio of £3445.56, and every increment of 1 AU in the KDQoL MCS is associated with an incremental cost-effectiveness ratio of £14.44. Assuming comparative effectiveness of the kidney BEAM intervention compared with in-person kidney rehabilitation,^7^^,^^27^ the average cost implication is £708/participant/yr for in-person rehabilitation compared to £15/participant/yr for delivery of the kidney BEAM intervention, a suggested cost saving of £693 per participant.

DHIs present a real opportunity for health care payers such as the NHS to deliver essential services where fiscal resources and workforce are not available to deliver face-to-face care. Furthermore, digital interventions offer convenience for patients who participate from home and choose when to exercise. The Kidney BEAM DHI is the first virtual solution in the kidney rehabilitation space to be proven to be cost-effective. Cost benefits of a similar magnitude have been realized with in-person and home-based exercise interventions in other long-term condition populations, such as people with cardiac and pulmonary conditions,^28^, ^29^, ^30^ and a recent systematic review revealed that cardiac rehabilitation DHIs were as cost effective as in-person cardiac rehabilitation.^31^ Kidney Beam has now been rolled-out across all 8 regions of England as part of an implementation project in preparation for commissioning. Results from the Kidney Beam Trial, together with practical experience gained through NHS implementation, will ensure that there is a clear plan for long-term adoption by the NHS. In addition, because the Kidney BEAM program is delivered online from a single center, it is simple to establish in a wide variety of health care systems and to offer to people across large geographical areas.

The Kidney BEAM physical activity DHI was developed using the Behavior Change Wheel methodology,^32^ a methodology based on 19 frameworks of behavior change theory, including the transtheoretical model of behavior change.^33^^,^^34^ Careful consideration and preparation of a logic model^18^ that incorporated key intervention functions to facilitate a change in behavior and overcome common barriers to engagement with physical activity^35^ was codeveloped with people with lived experience and experts in the field. The intention of the initial 12-week structured program of physical activity was to support people living with CKD to make important initial physiological and psychological gains in health outcomes to promote and sustain self-managed physical activity behavior after completion of the program. Evidence suggests that for meaningful behavior change to be achieved, there is a need for the “active” behavior to be maintained over a 6-month period.^36^ The Kidney BEAM intervention was deliberately designed to meet this expectation, combining the initial 12-week structured and supported physical activity DHI with a 12-week self-managed DHI component. This type of “kick-start” program has been successfully utilized in in-person kidney-specific rehabilitation^7^ as well as in-person physical rehabilitation for other chronic conditions^37^, ^38^, ^39^ and has resulted in a maintenance of health outcome gains and physical activity behavior in the longer term.^28^, ^29^, ^30^

The significant improvement we continue to report in the KDQoL MCS at 6-months was likely driven by changes in the KDQoL subscales of emotional wellbeing, role emotional, social function and vitality (energy or fatigue) scales, because these subscales are more heavily weighted in the calculation of the MCS score. However, the improved physical functioning, role physical, bodily pain, and general health scores were also all improved, so those subscales will also have contributed to the improvement in MCS score. It is noteworthy that improvements in mental HRQoL, patient activation, and physical function were realized at 12 weeks^14^ suggesting the BEAM platform “kick-started” improvements in HRQoL during the initial 12-week structured component of the intervention. It is encouraging to witness sustained and continued mental HRQoL gains with the self-managed physical activity component of the intervention, particularly in a patient population where lower patient activation levels have been recognized and are associated with a lower HRQoL in people living with CKD.^40^

The Kidney Beam Trial was inclusive of people living with CKD from across the disease trajectory, including predialysis and those people requiring dialysis treatment or living with a kidney transplant. Although it is acknowledged that the mental burden of symptoms associated with kidney disease, which vary along with disease stage and are highest among dialysis recipients,^41^ may be a challenge to treat with a one-size-fits-all physical activity DHI, the inclusion of a seated option and a standing option for performing the activity did allow for an inclusive approach; and the health coaching provided by the physiotherapy assistant encouraged a tailored approach to commencement and progression of the program for all participants. The baseline global physical activity questionnaire revealed a mean score of only 110 min/wk. The mean additional physical activity minutes recorded on the platform was 44 minutes at 12 weeks, and 22 minutes at 6-months, almost 50% and 25% increases, respectively. In addition, given that the global physical activity questionnaire may overestimate scores, the increase in physical activity as a result of the Kidney BEAM intervention is important, especially because even small increases in physical activity can have a major impact upon health outcomes for this patient population.^4^ An adherence rate of 63% with the 12-week “kick-start” program may be considered as moderate, but compared favorably with physical activity DHIs for other long-term conditions (55%)^42^ and face-to-face renal rehabilitation programs (59%).^27^ Although we aimed to encourage participant engagement with behavioral change techniques such as motivational interviewing, it is acknowledged that further work to personalize DHIs may lead to better engagement with these physical activity interventions.

A limitation of the trial was the restriction of the trial sites to a single country and delivery of the intervention in the English language only. Although the Kidney BEAM physical activity platform was deliberately codeveloped with people living with the condition, including people with generally poor digital literacy, people from lower socioeconomic backgrounds, minority ethnic groups and elderly patients, there is acknowledgement that further work is required to meet the needs of these populations who are expected to benefit the most from health promoting strategies in the setting of CKD, including DHIs. Substudies are underway to expand relevant content, translate the website into other languages and address digital literacy and access. These limitations may partially explain the limited recruitment rate observed in the Kidney BEAM trial and does mean that the generalizability of the trial findings to CKD populations worldwide will require further evaluation.

The primary and secondary outcomes were self-reported and because participants were not blinded to the allocated treatment, this method will have produced bias. We could not mask the supporting physiotherapy assistants. However, the health economist and statisticians were masked. Health care utilization for primary and social care were collected via patient interview, which may have introduced recall bias. Concurrent medication usage and sleep quality were not analyzed as part of this current trial, and it is acknowledged that these may affect mental HRQoL. There was a dropout rate of 39.8% from the intervention group at 6-months, which required data to be imputed and may increase imprecision in estimates. There was no obvious difference in participant characteristics between groups for complete and incomplete cases and over 75% of the dropouts were within the first week of the trial. The last observation carried forward approach to missing data generally offers a conservative estimate of the patient’s outcome trajectory in a study^43^; however, it can lead to an overestimation of the size of the effect of the intervention. Per protocol analyses were conducted to confirm the results.

Recruitment for this trial was during the COVID-19 pandemic, a time when recruitment to trials was particularly challenging, especially for more vulnerable patients (such as the elderly and those with comorbidities). This contributed to the slightly younger and less comorbid population we recruited. However, the study recruited a more diverse and representative population than previous exercise interventions.^44^ The inclusion of earlier CKD stages was a strength of this current study, because most health care systems do not have capacity to support these patients using traditional methods of face-to-face exercise intervention.

Participants from the waitlist control group were offered access to the kidney beam intervention at 12 weeks. We acknowledge that it would have been ideal to ask people from the waitlist control group to wait until 6-months to access the platform; however, because this randomized controlled trial was conducted during the COVID-19 pandemic, withholding access to a potentially useful intervention for promoting mental HRQoL was deemed unethical. Only 11% of people from this group chose to access the platform during this time; nevertheless, it is acknowledged that this may have led to an underestimation of the size of the effect between the Kidney BEAM group and the waitlist control group.

Overall, this trial demonstrates that the Kidney BEAM physical activity platform is a clinically beneficial and cost-effective DHI to improve mental HRQoL in people with CKD. The results provide evidence to support commissioning within the UK NHS.

## Disclosure

King’s College Hospital NHS Trust and SAG were involved in the conception and development of Kidney BEAM. SAG became a director of Kidney Beam Ltd in August 2023. SB was a previous Trustee of Kidney Research UK. DW has an ongoing consultancy contract with AstraZeneca and has received honoraria/consultancy fees from Astellas, Boehringer Ingelheim, Bayer, Eledon, Galderma, GlaxoSmithKline, Gilead, Janssen, Mundipharma, ProKidney, Tricida, Vifor, and Zydus for activities related to education and clinical trials. JC and NC were both independent contractors but were paid for by the grant. All the other authors declared no competing interests.

## Supplementary Material

Supplementary File (PDF) **Table S1.** Summary of QALYs. **Table S2.** Base-case model KDQol MCS. **Table S3.** Sensitivity analysis reporting different values for cost of the intervention per person. **Table S4.** Summary of observed costs by category, time-period, and intervention group. **Table S5.** Sources of resource use and unit costs. **Table S6.** Response of primary and secondary outcomes to the Kidney BEAM intervention (per protocol analyses). **Table S7.** Comparison of missing data between complete cases and missing cases due to trial dropouts. **Table S8.** Table of missingness in the data. CONSORT 2010 Checklist of information to include when reporting a randomized trial.

## Footnotes

Supplementary File (PDF)
**Table S1.** Summary of QALYs. **Table S2.** Base-case model KDQol MCS. **Table S3.** Sensitivity analysis reporting different values for cost of the intervention per person. **Table S4.** Summary of observed costs by category, time-period, and intervention group. **Table S5.** Sources of resource use and unit costs. **Table S6.** Response of primary and secondary outcomes to the Kidney BEAM intervention (per protocol analyses). **Table S7.** Comparison of missing data between complete cases and missing cases due to trial dropouts. **Table S8.** Table of missingness in the data. CONSORT 2010 Checklist of information to include when reporting a randomized trial.
