# Supplementary material for: Kidney Beam-A Cost-Effective Digital Intervention to Improve Mental Health
Source: Kidney Int Rep. 2024 Sep 2;9(11):3204–17. doi: 10.1016/j.ekir.2024.08.030 (PMC11551101; doi:10.1016/j.ekir.2024.08.030)
Supplement: Supplementary File (PDF) — Table S1. Summary of QALYs. Table S2. Base-case model KDQol MCS. Table S3. Sensitivity analysis reporting different values for cost of the intervention per person. Table S4. Summary of observed costs by category, time-period, and intervention group. Table S5. Sources of resource use and unit costs. Table S6. Response of primary and secondary outcomes to the Kidney BEAM intervention (per protocol analyses). Table S7. Comparison of missing data between complete cases and missing cases due to trial dropouts. Table S8. Table of missingness in the data. CONSORT 2010 Checklist of information to include when reporting a randomized trial. [file mmc1.pdf]

Supplementary Material

Supplementary data table 1: Summary of QALYs

	**Kidney beam**			**Waiting list**			**Kidney beam vs Waiting list**
	**N**	**Mean (SD)**	**Median (IQR)**	**N**	**Mean (SD)**	**Median (IQR)**	**Mean difference (95% confidence interval*)**
**EQ5D-3L (Quality adjusted life years)**							
0 months	92	0.675 (0.235)	0.730 (0.573 to 0.840)	135	0.728 (0.237)	0.790 (0.636 to 0.889)	-0.053 (-0.114 to 0.010)
12 weeks	92	0.710 (0.235)	0.740 (0.605 to 0.890)	135	0.723 (0.240)	0.743 (0.632 to 0.890)	-0.014 (-0.075 to 0.049)
6 months	83	0.803 (0.164)	0.805 (0.711 to 0.985)	118	0.664 (0.266)	0.728 (0.575 to 0.813)	0.139 (0.082 to 0.199)
0 to 6 months (AUC)	83	0.367 (0.096)	0.386 (0.312 to 0.445)	118	0.355 (0.115)	0.377 (0.310 to 0.432)	0.012 (-0.17 to 0.041)

***based on 5000 bootstrapped sample. AUC =Area under the EQ-5D curve**

Supplementary data table 2. Base case model KDQoL MCS (assumes intervention £15 per person per year)

	**Base case model: LVCF for missing cost components adjusted for baseline costs and MCS**
N: WL<br>N: KB	132⁺<br>91⁺
Mean difference in Cost	£139.50<br>(-£320.30 to £678.00)
Mean difference in MCS	9.66<br>(7.66 to 11.65)

Incremental cost effectiveness ratio (ICER)	£14.44

Calculated at the average baseline value of cost (£1850) and MCS score (45)

⁺ Excludes individuals with missing EQ-5D and cost baseline data (4 WL, 2 KB)

Supplementary Table 3. Sensitivity analysis reporting different values for cost of the intervention per person

	**Intervention cost £30 per year**	**Intervention cost £50 per year**	**Intervention cost £100 per year**
N: WL<br>N: KB	135<br>92	135<br>92	135<br>92
Mean difference in Cost	£98.10<br>(-£343.30 to £603.90)	£103.70<br>(-£327.20 to £596.40.90)	£120.20<br>(-£314.60 to £622.60)
Mean difference in QALYs	0.027<br>(0.012 to 0.040)	0.027<br>(0.013 to 0.040)	0.027<br>(0.013 to 0.041)
Incremental Cost effectiveness ratio (ICER)	£3,633.33	£3,840.74	£4,451.85
Probability CE @ £20,000 per QALY gained	0.93	0.93	0.92
Probability CE @ £30,000 per QALY gained	0.98	0.98	0.98

Supplementary data table 4. Summary of observed costs by category, time period and intervention group

	Costs (UK£)						
	Kidney beam			Waiting list			Waiting list vs Kidney beam
	N	Mean (SD)	Median (IQR)	N	Mean (SD)	Median (IQR)	Mean Cost difference (95% confidence interval*)
**12 weeks prior to intervention**							
Inpatient	93	572.31 (1984.40)	0.00 (0.00 to 0.00)	136	789.12 (2112.72)	0.00 (0.00 to 0.00)	-216.81 (-739.74 to 339.08)
Outpatient	92	653.05 (1789.46)	181.54 (181.54 to 544.61)	135	745.66 (3239.33)	363.07 (181.54 to 544.61)	-92.61 (-819.35 to 511.55)
A&E	93	40.24 (111.11)	0.00 (0.00 to 0.00)	135	29.48 (105.04)	0.00 (0.00 to 0.00)	10.77 (-17.48 to 39.06)
GP & primary care	93	53.41 (69.88)	26.00 (6.80 to 69.21)	136	49.90 (62.88)	37.70 (6.80 to 61.80)	3.51 (-13.71 to 21.33)
Social Services	93	0.81 (5.77)	0.00 (0.00 to 0.00)	135	2.40 (14.65)	0.00 (0.00 to 0.00)	-1.59 (-4.62 to 0.84)
Medication	93	6.15 (23.17)	0.00 (0.00 to 0.76)	133	12.27 (34.30)	0.00 (0.00 to 2.92)	-6.12 (-13.92 to 1.21)
**0 to 12 weeks intervention period**							
Inpatient	93	106.97 (725.45)	0.00 (0.00 to 0.00)	134	272.32 (1317.23)	0.00 (0.00 to 0.00)	-165.34 (-431.12 to 100.43)
Outpatient	92	692.75 (3933.39)	181.54 (0.00 to 363.15)	135	607.91 (3238.91)	181.54 (0.00 to 363.07)	84.84 (-837.79 to 1167.00)
A&E	93	25.87 (97.07)	0.00 (0.00 to 0.00)	136	36.00 (120.53)	0.00 (0.00 to 0.00)	-10.13 (-38.93 to 19.31)
GP & primary care	88	49.81 (81.96)	16.70 (0.00 to 65.95)	135	46.04 (69.77)	33.00 (0.00 to 55.61)	3.77 (-16.49 to 24.71)
Social Services	91	11.55 (53.23)	0.00 (0.00 to 0.00)	133	7.00 (49.50)	0.00 (0.00 to 0.00)	4.56 (-8.76 to 18.59)

Medication	83	3.25 (9.13)	0.00 (0.00 to 1.46)	12 2	6.49 (19.54)	0.00 (0.00 to 1.46)	-3.25 (-7.68 to 0.47)
**12 weeks to 6 months intervention period**							
Inpatient	88	490.99 (1738.94)	0.00 (0.00 to 0.00)	13 6	122.53 (821.16)	0.00 (0.00 to 0.00)	368.45 (-19.67 to 756.58)
Outpatient	89	212.21 (359.81)	0.00 (0.00 to 181.54)	13 4	376.08 (781.46)	181.54 (0.00 to 363.07)	-163.87 (-320.66 to -17.14)
A&E	89	35.02 (116.50)	0.00 (0.00 to 0.00)	13 3	43.76 (117.61)	0.00 (0.00 to 0.00)	-8.75 (-39.38 to 24.90)
GP & primary care	88	31.14 (47.53)	0.00 (0.00 to 42.00)	13 2	43.64 (82.92)	6.80 (0.00 to 42.00)	-12.50 (-30.63 to 4.60)
Social Services	89	0.28 (2.65)	0.00 (0.00 to 0.00)	13 4	2.93 (33.95)	0.00 (0.00 to 0.00)	-2.65 (-9.15 to 0.75)
Medication	82	3.48 (11.00)	0.00 (0.00 to 1.29)	13 3	7.98 (36.16)	0.00 (0.00 to 0.00)	-4.50 (-11.75 to 1.17)

*** based on 5000 bootstrapped sample. T**able summarises data on Inpatient, A&E, Outpatient, GP & primary care, Social Services, and Medication by intervention group at 12 weeks prior, and 12 weeks during the intervention. The results show a large amount of variation in the costs incurred within intervention groups (indicated by the large standard deviation) and the right-skewed (non-normal) distribution of the cost data (indicated by the median being less than the mean).

Supplementary Data Table 5: Sources of resource use and unit costs (UK£2021/22)

	**Value**	**Source**
**Hospital costs**		
A&E	£267.34	https://www.england.nhs.uk/costing-in-the-nhs/national-cost-collection/
Ambulance	£236.81	
Non-elective inpatient – long term	£4408.91	

Non-elective inpatient – short term	£801.11	
Elective inpatients	£5845.22	
Day case	£1038.36	
Outpatient attendance	£182	
Medication costs		British National Formulary 2022 https://www.bnf.org/products/bnf-online/
**Primary and community health visits**		
GP appointment	£42.00	Personal Social Services Research Unit. Unit costs of health and social care 2022 https://kar.kent.ac.uk/100519/1/Unit_Costs_of_Health_and_Social_Care_2022
Practice Nurse appointment	£13.00	
Walk-in centre	£27.50	
Counsellor / Mental health	£33.00	
Phone call with pharmacist	£11.00	
**Social Support Services**		
Social worker	£50 per hour	Personal Social Services Research Unit. Unit costs of health and social care 2012. https://kar.kent.ac.uk/100519/1/Unit_Costs_of_Health_and_Social_Care_2022
Home help/ care worker	£23 per hour	
Community physiotherapist	£144 per hour	
Community occupational health	£118 per hour	

Physiotherapy assistant time – health coaching	£12.24 per hour	https://kar.kent.ac.uk/100519/1/Unit_Costs_of_Health_and_Social_Care_2022
**6-month digital health intervention**		
Physical Activity DHI (inclusive of health coaching from Physiotherapy Assistant, Senior Physiotherapist oversight, platform running costs)	£15 subscription fee per participant (based on a 10% population usage contract for NHS England regions)	

.

Supplementary data table 6. Response of primary and secondary outcomes to the Kidney BEAM intervention (per protocol analyses)

	**n**	**Baseline** mean (SD)	**6 months** mean (SD)	**Mean difference in change between groups (kidney BEAM - waitlist control)** mean {95% CI}	**p value**	**Observed power**

**Primary outcome**						
**KDQoL MCS (AU)**						
Kidney BEAM	75	46.0 (10.2)	53.3 (7.8)	9.8 (7.5-12.2)	<.0001	1.00
Waitlist control	106	46.1 (10.5)	43.4 (10.6)			
**Secondary outcomes**						
**KDQOL PCS (AU)**						
Kidney BEAM	75	41.3 (11.9)	47.6 (8.2)	5.4 (3.3-7.4)	<.0001	1.00
Waitlist control	106	40.4 (11.1)	41.4 (11.0)			
**Symptom problem list**						
Kidney BEAM	96	77.0 (17.9)	79.6 (16.9)	1.6 (2.7-5.9)	0.465	0.113
Waitlist control	67	80.6 (15.5)	79.9 (19.2)			
**Effects of Kidney Disease**						
Kidney BEAM	82	70.2 (25.7)	78.0 (22.5)	5.1 (0.03,10.2)	0.049	0.506
Waitlist control	114	76.1 (22.5)	76.2 (25.5)			
**Burden of kidney disease**						
Kidney BEAM	76	56.2 (30.5)	68.3 (26.6)	10.3 (5.8-14.9)	<.0001	.994
Waitlist control	106	64.4 (29.5)	64.6 (28.5)			
**Work status**						
Kidney BEAM	84	61.8 (40.6)	61.6 (38.1)	-5.2 (-12.3-2.0)	0.155	0.295
Waitlist control	120	61.7 (41.4)	65.8 (37.8)			
**Cognitive function**						
Kidney BEAM	45	74.5 (19.6)	81.9 (15.0)	7.8 (2.6-12.9)	0.0034	0.844
Waitlist control	58	81.1 (17.9)	78.6 (20.2)			
**Quality of social interaction**						
Kidney BEAM	76	72.7 (17.8)	82.6 (13.2)	14.7 (10.4-19.0)	<.0001	1.00
Waitlist control	108	74.5 (16.3)	65.0 (19.0)			
**Sexual function**						
Kidney BEAM	44	38.1 (41.8)	42.8 (40.7)	-2.6 (-16.0-10.8)	0.701	0.067
Waitlist control	67	50.9 (43.6)	44.4 (45.0)			
**Sleep**						

Kidney BEAM	85	55.4 (17.9)	65.6 (13.9)	12.3 (8.3-16.2)	<.0001	1.00
Waitlist control	120	58.3 (20.1)	55.3 (21.0)			
**Social support**						
Kidney BEAM	73	74.5 (28.2)	81.5 (23.1)	10.9 (3.4-18.4)	0.0047	0.813
Waitlist control	98	74.7 (29.4)	71.6 (30.3)			
**Dialysis staff encouragement**						
Kidney BEAM	28	86.6 (18.3)	76.04 (28.1)	-13.5 (-25.7- -1.3)	0.031	0.587
Waitlist control	38	77.0 (28.4)	81.0 (27.2)			
**Overall health**						
Kidney BEAM	85	60.1 (19.9)	62.6 (7.9)	-1.3 (-5.1-2.9)	0.549	0.092
Waitlist control	118	58.1 (18.1)	61.7 (20.8)			
**Patient satisfaction**						
Kidney BEAM	34	72.5 (22.4)	72.6 (22.8)	-0.2 (-8.6-8.1)	0.959	0.050
Waitlist control	45	70.7 (27.8)	74.3 (24.2)			
**Physical functioning**						
Kidney BEAM	84	65.3 (29.4)	78.4 (20.7)	13.0 (8.5-17.4)	<.0001	1.00
Waitlist control	120	62.7 (29.8)	63.3 (29.4)			
**Role physical**						
Kidney BEAM	85	50.9 (42.4)	79.9 (35.5)	24.4 (14.8-34.1)	<.0001	1.00
Waitlist control	120	49.4 (42.9)	54.0 (43.6)			
**Pain**						
Kidney BEAM	89	62.3 (26.2)	72.5 (23.5)	10.3 (5.8-14.9)	<.0001	.994
Waitlist control	127	67.4 (27.4)	62.0 (30.1)			
**General health**						
Kidney BEAM	75	43.0 (21.2)	53.0 (19.4)	11.1 (7.0-15.2)	<.0001	1.00
Waitlist control	106	41.9 (20.2)	41.1 (21.2)			
**Emotional wellbeing**						
Kidney BEAM	76	69.5 (18.6)	83.1 (13.9)	18.7 (15.1-22.4)	<.0001	1.00
Waitlist control	105	71.0 (17.6)	65.2 (20.0)			
**Role emotional**						

Kidney BEAM	85	64.7 (41.3)	86.2 (30.3)	20.8 (10.5-31.1)	<.0001	0.98
Waitlist control	120	62.5 (41.8)	64.2 (43.2)			
**Social function**						
Kidney BEAM	76	67.9 (25.2)	82.1 (20.2)	18.3 (13.2-24.5)	<.0001	1.00
Waitlist control	107	62.6 (29.9)	59.2 (29.1)			
**Energy/fatigue**						
Kidney BEAM	75	43.2 (20.6)	64.4 (18.7)	27.4 (23.5-31.4)	<.0001	1.00
Waitlist control	106	44.8 (23.0)	37.8 (22.6)			
**EQ-5D-3L utility score**			`			
Kidney BEAM	83	0.69 (0.22)	0.81 (0.16)	0.16 (0.13-0.20)	<.0001	1.00
Waitlist control	120	0.72 (0.23)	0.66 (0.26)			

Data are mean (standard deviation), median (interquartile range), or mean {95% confidence interval} ANCOVA adjusted scores. Control, waitlist control group; Kidney BEAM, Kidney BEAM intervention group (physical activity training and education plus usual care); KDQOL, Kidney Disease Quality of Life Short Form (KDQOL-SF 1.3); PCS, Physical Component Summary; AU, arbitrary units; EQ-5D-3L, EuroQol five-dimension descriptive system.

Supplementary Data Table 7. Comparison of missing data between complete cases and missing cases due to trial dropouts

	**Complete cases**				**Lost to follow up / withdrew cases**			
	**Kidney BEAM (n=105)**		**Waitlist control (n=142)**		**Kidney BEAM (n=68)**		**Waitlist control (n=25)**	
	N or mean	% or SD	N or mean	% or SD	N or mean	% or SD	N or mean	% or SD
**Sex - no. (%)**								
Male	53	50	66	46	43	63	13	52
Female	52	50	74	52	25	37	12	48
**Age - years**	55	14	54	14	53	13	49	13

**Ethnicity - no. (%)**								
Black	14	13	15	18	6	9	4	16
White	77	73	109	77	50	74	18	72
Asian	11	10	15	11	11	16	2	8
Other	3	3	2	1	1	1	1	4
**Stage of CKD – no. (%)**								
Stage 2	15	14	25	18	12	18	3	12
Stage 3A	17	16	20	14	12	18	13	52
Stage 3B	31	30	28	20	14	21	3	12
Stage 4	21	20	31	22	13	19	2	8
Stage 5	20	19	38	27	17	25	4	16
**Comorbidities – no. (%)**								
Cardiovascular accident	2	2	4	3	6	10	0	0
Myocardial Infarction	2	2	5	3	1	2	0	0
Diabetes Mellitus	21	20	36	25	16	24	3	12
Hypertension	69	66	105	74	46	68	10	40

Supplementary Data Table 8: Table of missingness in the data

	**Waiting list**		**Kidney BEAM**	
	**Complete data**	**Missing observations**	**Complete data**	**Missing observations**
**At baseline (pre-12 weeks)**	N=167		173	
Inpatients	167	0	173	0
A&E	166	1	173	0
Outpatients	166	1	173	1

GP & primary care	166	1	173	0
Social services	166	1	173	0
Medication	164	3	173	0
EQ-5D	166	1	171	3
Total costs	164	3	173	1
Total costs & EQ5D	163	4	170	4
**At 12 weeks**	N=155		N=105	
Inpatients	153	2	105	0
A&E	155	0	104	1
Outpatients	154	1	104	1
GP & primary care	154	1	99	6
Social services	152	3	102	3
Medication	140	15	94	11
EQ-5D	153	2	104	1
Total costs	134	21	89	16
Total costs & EQ5D	132	26	88	18
**At 26 weeks**	N=136		N=93	
Inpatients	136	0	88	5
A&E	136	0	91	2
Outpatients	134	2	89	4
GP & primary care	132	4	88	5
Social services	134	2	89	4
Medication	133	3	82	11
EQ-5D	118	18	83	10
Total costs	128	8	80	13
Total costs & EQ5D	110	26	75	18

CONSORT 2010 checklist of information to include when reporting a randomised trial*

Section/Topic	Item No	Checklist item	Reported on page No
**Title and abstract**			
	1a	Identification as a randomised trial in the title	1
	1b	Structured summary of trial design, methods, results, and conclusions (for specific guidance see CONSORT for abstracts)	4
**Introduction**			
Background and objectives	2a	Scientific background and explanation of rationale	5-6
	2b	Specific objectives or hypotheses	6
**Methods**			
Trial design	3a	Description of trial design (such as parallel, factorial) including allocation ratio	15
	3b	Important changes to methods after trial commencement (such as eligibility criteria), with reasons	N/A
Participants	4a	Eligibility criteria for participants	15
	4b	Settings and locations where the data were collected	15
Interventions	5	The interventions for each group with sufficient details to allow replication, including how and when they were actually administered	17
Outcomes	6a	Completely defined pre-specified primary and secondary outcome measures, including how and when they were assessed	16-17
	6b	Any changes to trial outcomes after the trial commenced, with reasons	N/A
Sample size	7a	How sample size was determined	18
	7b	When applicable, explanation of any interim analyses and stopping guidelines	N/A
Randomisation:			
Sequence generation	8a	Method used to generate the random allocation sequence	16
	8b	Type of randomisation; details of any restriction (such as blocking and block size)	16
Allocation concealment mechanism	9	Mechanism used to implement the random allocation sequence (such as sequentially numbered containers), describing any steps taken to conceal the sequence until interventions were assigned	16
Implementation	10	Who generated the random allocation sequence, who enrolled participants, and who assigned participants to interventions	16
Blinding	11a	If done, who was blinded after assignment to interventions (for example, participants, care providers, those assessing outcomes) and how	16
	11b	If relevant, description of the similarity of interventions	N/A
Statistical methods	12a	Statistical methods used to compare groups for primary and secondary outcomes	18-20
	12b	Methods for additional analyses, such as subgroup analyses and adjusted analyses	18-20

Section/Topic	Item No	Checklist item	Reported on page No
**Results**			
Participant flow (a diagram is strongly recommended)	13a	For each group, the numbers of participants who were randomly assigned, received intended treatment, and were analysed for the primary outcome	23
	13b	For each group, losses and exclusions after randomisation, together with reasons	23
Recruitment	14a	Dates defining the periods of recruitment and follow-up	6
	14b	Why the trial ended or was stopped	6
Baseline data	15	A table showing baseline demographic and clinical characteristics for each group	25
Numbers analysed	16	For each group, number of participants (denominator) included in each analysis and whether the analysis was by original assigned groups	18-20
Outcomes and estimation	17a	For each primary and secondary outcome, results for each group, and the estimated effect size and its precision (such as 95% confidence interval)	8-10
	17b	For binary outcomes, presentation of both absolute and relative effect sizes is recommended	8-10
Ancillary analyses	18	Results of any other analyses performed, including subgroup analyses and adjusted analyses, distinguishing pre-specified from exploratory	8-10
Harms	19	All important harms or unintended effects in each group (for specific guidance see CONSORT for harms)	9-10
**Discussion**			
Limitations	20	Trial limitations, addressing sources of potential bias, imprecision, and, if relevant, multiplicity of analyses	14
Generalisability	21	Generalisability (external validity, applicability) of the trial findings	14
Interpretation	22	Interpretation consistent with results, balancing benefits and harms, and considering other relevant evidence	14
**Other information**			
Registration	23	Registration number and name of trial registry	20
Protocol	24	Where the full trial protocol can be accessed, if available	20
Funding	25	Sources of funding and other support (such as supply of drugs), role of funders	20

Citation: Schulz KF, Altman DG, Moher D, for the CONSORT Group. CONSORT 2010 Statement: updated guidelines for reporting parallel group randomised trials. BMC Medicine. 2010;8:18. © 2010 Schulz et al. This is an Open Access article distributed under the terms of the Creative Commons Attribution License (http://creativecommons.org/licenses/by/2.0), which permits unrestricted use, distribution, and reproduction in any medium, provided the original work is properly cited.

*We strongly recommend reading this statement in conjunction with the CONSORT 2010 Explanation and Elaboration for important clarifications on all the items. If relevant, we also recommend reading CONSORT extensions for cluster randomised trials, non-inferiority and equivalence trials, non-pharmacological treatments, herbal interventions, and pragmatic trials. Additional extensions are forthcoming: for those and for up-to-date references relevant to this checklist, see www.consort-statement.org.
